# A randomized controlled, trial on effects of mobile phone text messaging in combination with motivational interviewing versus standard infant feeding counselling on breastfeeding and child health outcomes, among women living with HIV

**DOI:** 10.1186/s13006-024-00693-2

**Published:** 2025-01-20

**Authors:** Moleen Zunza, Lehana Thabane, Louise Kuhn, Christine Els, Carl Lombard, Mark F. Cotton, Taryn Young

**Affiliations:** 1 Division of Epidemiology & Biostatistics, Faculty of Medicine and Health Sciences, Stellenbosch University, Francie van Zijl Drive, PO Box 241, Cape Town, 8000 South Africa; 2 Department of Biostatistics Health Research Methods, Evidence, and Impact, McMaster University, Hamilton, Canada; 3 Biostatistics Unit, Father Sean O’Sullivan Research Centre, St. Joseph’s Healthcare, Toronto, Canada; 4 Gertude H. Sergievsky Center, Vagelos College of Physicians and Surgeons, Columbia University Irving Medical Center, New York, USA; 5 Western Cape Department of Health, Khayelitsha District Hospital, Khayelitsha, South Africa; 6 Department of Paediatrics and Child Health, Family Center for Research with Ubuntu, Stellenbosch University, Cape Town, South Africa

**Keywords:** Breastfeeding, Breastfeeding exclusive, Exclusive breastfeeding, HIV/AIDS, Mobile phone text messaging, Motivational interviewing

## Abstract

**Background:**

Despite efforts to promote optimal breastfeeding practices, the practice of exclusive breastfeeding is low in South Africa. We conducted a trial to determine whether text messaging plus motivational interviewing prolonged exclusive breastfeeding during the first six months of life and improved child health outcomes.

**Methods:**

We conducted a randomized parallel group-controlled trial between July 2022 and May 2024, at a secondary-level healthcare facility. Mothers living with HIV, 18 years or older, initiating breastfeeding, on combination antiretroviral therapy (cART) and their infants were enrolled. The primary endpoint was exclusive breastfeeding from birth through week 24, based on the consecutive 24-hour food recall interviews. We compared differences in exclusive breastfeeding rates using a proportion test. Mothers who completely stopped breastfeeding were asked an open-ended question on reasons for stopping breastfeeding.

**Results:**

Using block randomization mother-child pairs (*n* = 276) were randomly allocated to receive intervention (*n* = 138) or standard infant feeding counselling (*n* = 138), of whom 105 and 101 mother-child pairs in the intervention group and standard care group, respectively, completed all four study visits. Exclusive breastfeeding rate at 24 weeks in the intervention group was 6% (6/105) and 7% (7/101) in the standard care group, rate difference − 1% (95% CI -6–4%). Sixty-two of 276 mothers completely stopped breastfeeding, of whom 25% (34/138) and 20% (28) were in the intervention group and standard care group, respectively. The most common reasons for stopping breastfeeding were the mother needing to return to work or look for work, 66% (*n* = 41). We also found that early breastfeeding cessation increased risk of child hospitalization or death compared to any form of breastfeeding to week 24, 10% (5/48) versus 3% (5/158), *p* = 0.055.

**Conclusions:**

We found no effect of the intervention on exclusive breastfeeding rates. Early cessation of breastfeeding was prevalent and maternal employment characteristics are important social determinants of breastfeeding behaviour. There is need for further research evaluating the effect of interventions that include financial incentives on breastfeeding practices among socioeconomically disadvantaged mothers. HIV services should reliably offer cART, consistently monitor viral load, and support mothers cART adherence, in settings where mixed feeding is common.

**Trial registration:**

The trial was registered on ClinicalTrials.gov (NCT05063240) and Pan African Clinical Trial Registries (PACTR202110870407786) before recruitment of the first subject.

## Background

Breastfeeding is essential for preventing the triple burden of malnutrition, infectious diseases, and mortality, in low-income and high-income countries [1, 2]. The World Health Organization (WHO) recommends exclusive breastfeeding for the first six months of life, followed by continued breastfeeding with appropriate complementary foods for two years or longer [3, 4]. South Africa has made remarkable progress in reducing the risk of vertical transmission of HIV during the first 2 months of life, from 23% in 2003 to 0.7% in 2019 [5]. This success is largely attributed to the adoption of the “Option B +” strategy into policy, where mothers living with HIV receive combination antiretroviral treatment (cART), irrespective of CD4 count or clinical disease severity, to be maintained either for the duration of breastfeeding or as lifelong treatment [4]. In 2022, HIV prevalence among pregnant women in South was 25% and 99% of these women were on ART [6]. Given the benefits of breastfeeding and risks of not breastfeeding, mothers need to be supported to breastfeed their infants, while maintaining virological suppression to minimize the vertical transmission risk [5].

Over the past decade, exclusive breastfeeding among infants less than 6 months increased to 49% in 2019 in low-income countries [7]. Despite these important improvements, there are very few countries on track to meet the World Health Assembly target of 70% of infants being exclusively breastfed by 2030, and there are still large disparities between and within countries [8]. In South Africa exclusive breastfeeding rates in general population are at 32% [7] and 8–30% among mothers living HIV [8], due to provincial disparities [9].

Despite the counselling on infant-feeding offered at primary healthcare facilities and many interventions to promote optimal infant feeding, practices remain suboptimal [10, 11]. In South Africa, birthing mothers are referred at discharge to a community health worker to provide breastfeeding support post-delivery through home visits in addition to formal engagement with the health services. Mothers are counselled on infant feeding by primary healthcare nurses and trained lay counsellors during routine healthcare visits [12]. Studies showed that infant feeding counselling improves infant feeding practices in South Africa [13]. Mobile phone text messaging, a simple, low-cost intervention, improves medication adherence among patients with HIV, diabetes, and tuberculosis [14–16]. Motivational interviewing, a patient-centred non-coercive approach [17], is beneficial across many health problems, including weight loss, and medication adherence and retention in care among patients with HIV [18, 19]. There are different factors at multiple levels preventing mothers from following recommended infant feeding practices, leading to early breastfeeding cessation or mixed feeding during the first 6 months, particularly in low- and middle-income countries [20]. Infant feeding cultural norms, stigma, inadequate counselling and limited financial resources, and infant feeding knowledge are among the factors influencing infant feeding choices of mothers living with HIV [21–23].

Significant gaps still exist in developing innovative strategies to support breastfeeding in environments with low exclusive breastfeeding rates. The trial objective was to demonstrate the superiority of mobile phone text messaging in combination with motivational interviewing over standard infant feeding counselling in increasing week 24 exclusive- and any form of -breastfeeding rates, reducing risk of child hospitalization or death and improving linear growth.

## Methods

### Trial design

We previously described the study methods [24]. Briefly, we conducted a parallel group, standard care-controlled randomized trial evaluating infant feeding practices at four follow up visits, among 276 mother-child pairs. We randomly assigned participants to either weekly mobile phone text messaging plus in-person motivational interviewing or to standard infant counselling for 24 weeks. The principal investigator used Stata 17 random number generating command (RALLOC), to generate the randomization allocation sequence restricted by permuted block sizes of 2, 4 and 6, with a 1:1 allocation ratio. A research assistant enrolled study participants, collected baseline data using the Research Electronic Data Capture (REDCap) mobile application and uploaded the data to the REDCap online server. The principal investigator assigned study group sequentially, using the locked unmodifiable allocation sequence stored on the REDCap online server. An independent data analyst performed unblinded interim analysis during trial monitoring. Study participants and the principal investigator were aware of the group assignment. A research assistant not administering the study interventions completed outcome evaluation questionnaires at follow up visits, without knowledge of group assignment. This manuscript was written following the Consolidated Standards of Reporting Trials (CONSORT 2010) guidelines for reporting parallel group randomized trial [25].

### Setting and study population

Mothers were counselled to exclusively breastfeed for the first 6 months by nurses and trained lay counsellors during routine postnatal clinic visits at their local primary healthcare clinics. Khayelitsha District Hospital provides secondary-level healthcare services. Mothers were informed about the study and invited to participate within 24 h of giving birth at Khayelitsha District Hospital and followed for 6 months at Masiphuhlisane Research Centre in Khayelitsha, Cape Town. We enrolled mothers living with HIV and on cART, initiating breastfeeding, 18 years or older, with a mobile phone, and their infants. We excluded mothers who initiated formula feeding or who were advised by healthcare providers not to breastfeed due to high viral load or other breastfeeding contraindications, gave birth to more than one infant, infant birthweight < 2500 g or gestational age at birth < 36 weeks.

### Study interventions

#### Mobile phone text messaging and motivational interviewing

A research assistant sent text message weekly to mothers in the intervention group, encouraging exclusive breastfeeding and inquired if there were any breastfeeding problems. The research assistant contacted mothers who indicated a breastfeeding problem and those who failed to respond within 48 h. Text messages were dispatched weekly throughout the entire 6-month follow-up period, with disruptions only during the Christmas holiday breaks, between 15 December 2022 and 10 January 2023 and between 15 December 2023 and 10 January 2024, for mothers who were actively being followed up during that time. Mothers had face-to-face individual motivational interviews post-delivery at week 2, 6, and 10. During the interviews, the research assistant and the mother discussed breastfeeding practices and problems, potential solutions, reinforced mother’s own self-motivational statements and readiness to correct suboptimal infant feeding practices and affirm the mother’s freedom of choice. The discussions included the importance of exclusive breastfeeding, adherent to ART to maintain a suppressed viral load and risks of mixed feeding. Mothers who had problems with breastfeeding or were concerned about their viral load were advised to seek healthcare services at their primary health facility. Text messaging and motivational interviews were discontinued for mothers who completely stopped breastfeeding before trial conclusion. The mother-child pair who stopped breastfeeding early were followed for the secondary outcomes.

#### Standard care infant feeding counselling

As part of standard care mothers were referred to a community health worker to support ART adherence, and breastfeeding postdelivery, were expected to attend monthly routine child growth monitoring, receive infant feeding counselling by primary healthcare nurses and trained lay counsellors during routine visits. Maternal viral load monitoring was done at delivery and 6-monthly or every 4 to 6 weeks for mothers with suppressed and unsuppressed viral load, respectively. Babies were tested for HIV at birth, 6, 10 weeks and 6 months. Mothers’ attendance of standard of care routine visits was not monitored during the study.

#### Sample size, power, and detectable differences

The study was only powered to detect minimum importance difference exclusive breastfeeding rates between study groups. We expected exclusive breastfeeding rate of 8% from birth through 6 months in the standard care group [9]. To detect a difference of 15% between the intervention and standard care groups (i.e., 23% vs. 8%), 182 mother-infant pairs were required for a two-sided test traditional fixed sample size computation. Adjusting the sample size for two planned analyses using the O’Brien-Fleming inflation factor 1.01 at the 0.05 significance level and 80% power, we revised the sample size to 182 × 1.01 = 184 and further inflated the sample size by 33% to 275 mother -infant pairs, accounting for loss to follow up.

#### Study measurements and procedures

The study included an enrolment maternal interview at Khayelitsha District hospital, four in-person follow-up visits at week 2, 6, 10, and 24, at Masiphuhlisane Research Centre, child medical record review, and child length and weight measurements. Baseline sociodemographic and clinical characteristics were obtained by interviewing the mother and abstracting medical records data. At each visit, a research assistant interviewed the mother using a questionnaire of food items given to the child in the last week or 24 h preceding the inquiry. Mothers who completely stopped breastfeeding where asked an open-ended question on reasons for stopping breastfeeding. Infant death, hospitalization, safety events and other study related data were obtained by interviewing the mother.

#### Baseline clinical characteristics

We classified the time of starting cART as prior to conception, during pregnancy or at delivery according to mother self-report or abstracted from the medical records. We abstracted from the medical records the most recent viral load and CD4 count at delivery. Mother’s disclosure of HIV status was classified as yes or no according to mother self-report. Baseline socio-demographic characteristics were obtained by interviewing the mother.

### Study endpoints

#### Primary endpoints

The primary endpoints included exclusive breastfeeding and any form of breastfeeding from birth through week 24, based on the consecutive 24-hour food recall interviews. Exclusively breastfeeding was defined as a child who had only breastmilk and no other liquid or solid foods. Any form of breastfeeding was defined as a child who had breastmilk only or breastmilk and other liquid or solid foods. The infant feeding questionnaire was based on the WHO standardized instrument [26].

#### Secondary endpoints

Infant death or hospitalization for any cause, and non-routine or sick-clinic visits occurring within study duration, were obtained by interviewing the mother. Infant weight and length were measured by the research assistants.

#### Safety outcomes

Potential safety events of socially unintended consequences included relationship conflicts with the partner due to study participation, reduced child monitoring due to exaggerated perception of the benefits of breastfeeding (assessed infant immunization history as proxy), and inadvertent disclosure of participant’s HIV status.

### Statistical methods

We summarized baseline characteristics using descriptive statistics. We reported effect sizes with 95% confidence intervals. Statistical significance was set at *p* < 0.05.

### Primary analysis of primary endpoints

We conducted a complete-case analysis, including participants who completed all study follow-up visits for our primary analyses. A proportion test was used to compare differences in exclusive breastfeeding rates and any form of breastfeeding rates between study groups.

### Secondary analysis of primary endpoints

#### Binary endpoints

We conducted a binomial regression analysis on exclusive breastfeeding and any form of breastfeeding endpoints. Logistic regression was conducted to assess consistency of the effect.

### Analysis of secondary endpoints

#### Time to event endpoints

Analysis of time to stopping any form of breastfeeding and time to first all-cause hospitalization or death outcomes was compared between study groups using the log-rank test. Participants were censored at 24 weeks, or at last completed visit.

#### Binary endpoints

We compared the number of child hospitalization or death between study groups using chi-squared test and a logistic regression.

#### Continuous endpoints

WHO standardized weight-for-age, length-for-age and weight for-length z-scores were computed adjusting for gestational age at birth, using WHO Anthro Stata macro. We excluded z-scores below − 5 and above 5 from analysis. Mean weight-for-age, length-for-age and weight for-length z-scores were estimated and compared between study groups using random slope linear mixed models. The mixed effects model included study group, time and study group-time interaction as fixed effects and participant and time as random effects.

### Additional analysis imputing missing outcome data

We used multiple imputation to compute missing exclusive breastfeeding and any form of breastfeeding outcome data. We used birthweight, marital status (married versus not married), educational status (primary or no schooling versus secondary or tertiary) as predictor variables. We created 20 imputations using the Stata mi estimate: logit command that combines estimates using the Rubin’s pooling rules. Multiple imputation was done separately by study group.

### Interim analysis

The interim analysis was performed when 103 of the planned modified intention to treat sample size of 184 mother-child pairs completed the study. Of these, only 60 had complete data on the primary outcomes across all visits. The computed *z*-statistic based on 60 was 0.39, not exceeding the predefined O’Brien-Fleming stopping boundary value of ± 2.7967.

### Ethics considerations

Stellenbosch University Human Research Ethics committee (reference M21/03/010) approved the study. Western Cape Department of Health approved access to Khayelitsha District Hospital (reference WC_202107_007). The DSMB reviewed the unblinded safety data accruing in the study. The study provided transport to and from the study follow up research site; mothers received a R180 (∼ US $10) voucher at each study visit, as an incentive for their participation time.

## Results

**Fig. 1:**
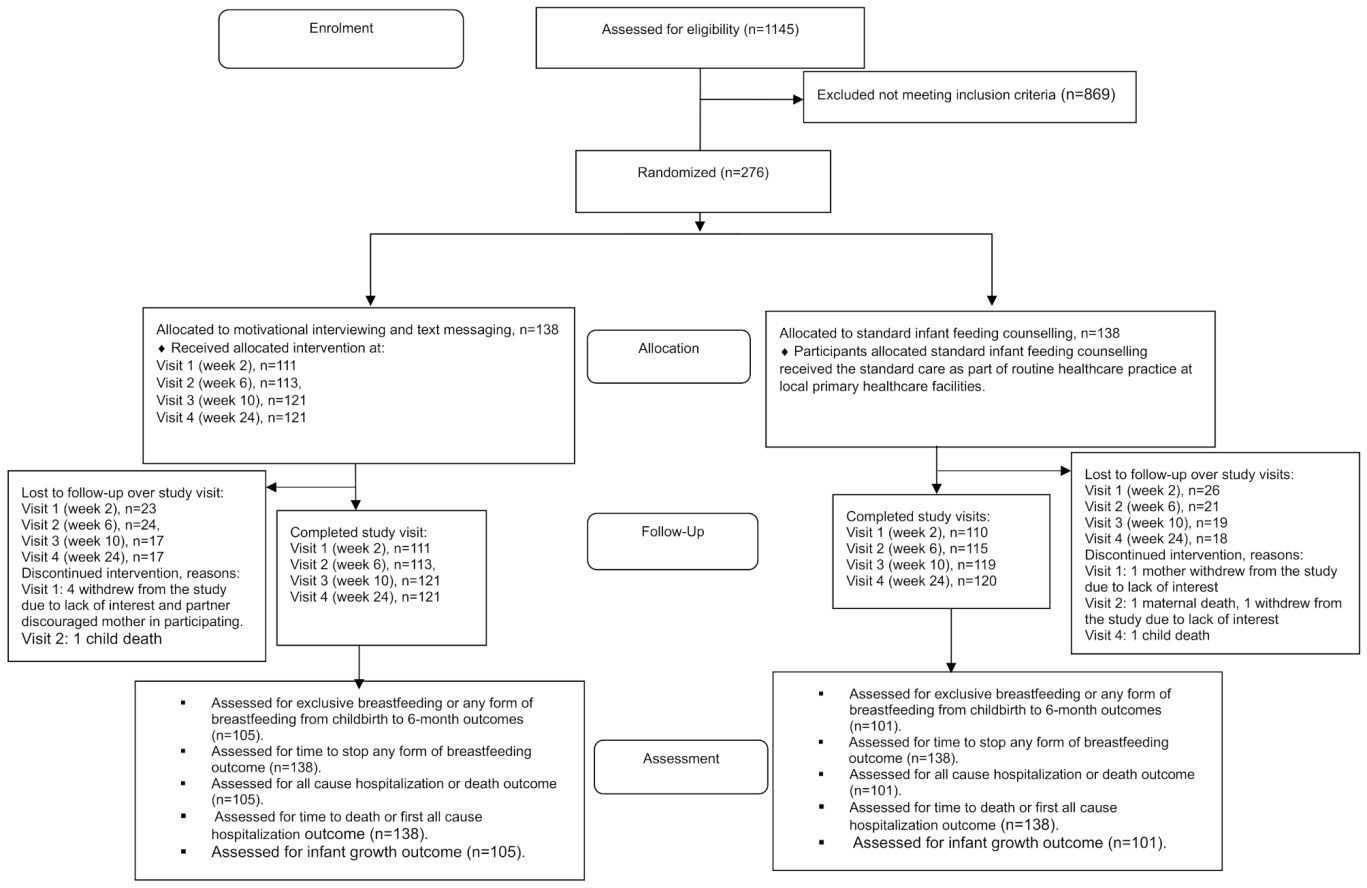
Flow of participants

Study recruitment started on 22 July 2022. We screened 1145 mother-infant pairs and recruited 276 mother-infant pairs by 27 November 2023. We randomly assigned 138 mother-infant pairs to each study group. We completed study follow up by 15 May 2024. Figure 1 shows participant flow. One hundred five and 101 participants in the intervention group and standard care group, respectively, had outcome data evaluation across all four study visits. Most mothers were unemployed, had high school education, were single or never married. Disclosure of HIV status to significant others was high, at 95%. Most (87%) mothers had HIV diagnosis before the index pregnancy (Table 1).

**Table 1:** Baseline demographic and clinical characteristics of study participants, *n* = 276

Characteristic	Intervention group, *n* = 138	Standard of care group, *n* = 138
Mother age, mean ± SD	32 ± 6 years	31 ± 6 years
Gestational age at booking, mean ± SD	16 ± 8 weeks	16 ± 8 weeks
Gestational age at delivery in weeks, mean ± SD	39 ± 1	40 ± 1
Mother most recent CD4 count, median (IQR)	481 (318 to 627) cells/µl	471 (302 to 702) cells/µl
Mother most recent viral load, median (IQR)	20 (20 to 44) copies/ml	20 (20 to 26) copies/ml
Birthweight, mean ± SD	3218 ± 416 g	3207 ± 383 g
Baby length, mean ± SD	50 ± 5 cm	50 ± 5 cm
Occupation, *n* (%)		
Unemployed Employed Student Other	87 (63%) 48 (35%) 2 (1%) 1 (1%)	91 (66%) 41 (30%) 4 (3%) 2 (1%)
Highest level of schooling, *n* (%) completed		
None completed Primary school High school Tertiary	0 (0%) 2 (1%) 131 (95%) 5 (4%)	2 (2%) 3 (2%) 126 (91%) 7 (5%)
Marital status, *n* (%)		
Single Married/living with partner Divorced/Widowed	79 (57%) 57 (41%) 2 (2%)	82 (59%) 56 (41%) 0 (0%)
Baby gender Girl, *n* (%)	69 (50%)	62 (45%)
Number of complete or incomplete pregnancies, *n* (%)		
1 2 3	23 (17%) 113 (82%) 2 (1%)	39 (28%) 97 (70%) 2 (2%)
Mode of delivery, *n* (%)		
Normal delivery Assisted normal delivery	137 (99%) 1 (1%)	136 (99%) 2 (1%)
Time of HIV diagnosis		
Before pregnancy During pregnancy At delivery	121 (88%) 16 (11%) 1 (1%)	120 (87%) 18 (13%) 0 (0%)
Time cART initiation, *n* (%)		
Before pregnancy During pregnancy At delivery	123 (89%) 14 (10%) 1 (1%)	118 (85%) 20 (15%) 0 (0%)
Disclosure of HIV status to significant others, *n* (%)		
No Yes	10 (7%) 128 (93%)	4 (3%) 134 (97%)
Baby on antiretroviral prevention, *n* (%)		
No Yes Don’t know	130 (94%) 4 (3%) 4 (3%)	131 (95%) 0 (0%) 7 (5%)

### Infant feeding practices

Exclusive breastfeeding practices were modest in both study groups through week 10 and dramatically dropped at week 24 (Table 2). We found no significant effect of the intervention on exclusive breastfeeding rates at week 24, (6% versus 7%), rate difference − 1% (95% CI -6–4%). After inputting missing outcome data, we found rate difference of -1% (95% CI -8–5%) (Table 3). The z-statistic of -0.36 did not exceed the predefined O’Brien-Fleming stopping boundary value of ± 1.9776.

**Table 2:** Estimate of breastfeeding rates at each study visit by study group

	Intervention group: *n* = 138		Standard care group: *n* = 138	
	*n*	Number of endpoints (%)	*n*	Number of endpoints (%)
Exclusive breastfeeding*				
Visit 1 (week 2)	111	65 (59%)	110	73 (66%)
Visit 2 (week 6)	113	67 (59%)	115	59 (51%)
Visit 3 (week 10)	121	56 (46%)	119	41 (34%)
Visit 4 (week 24)	121	13 (11%)	120	10 (8%)
Any form of breastfeeding*				
Visit 1 (week 2)	111	107 (96%)	110	108 (98%)
Visit 2 (week 6)	113	105 (93%)	115	110 (96%)
Visit 3 (week 10)	121	110 (91%)	119	107 (90%)
Visit 4 (week 24)	121	87 (72%)	120	92 (77%)

*Exclusive breastfeeding and any form of breastfeeding at each study visit based on 24-hour food recall interviews

**Table 3:** Estimate of breastfeeding rates, complete case analysis

	Intervention group: *n* = 138		Standard care group: *n* = 138		*p*-value	Rate difference (95% CI)
	*n*	Number of endpoints (%)	*n*	Number of endpoints (%)		
Co-primary outcomes*						
Exclusive breastfeeding from childbirth to 24 weeks	**105**	**6 (6%)**	**101**	**7 (7%)**	**0.72**	**-0.01(-0.06 to 0.04)**
Any form from breastfeeding from childbirth to 24 weeks	**105**	**79 (75%)**	**101**	**79 (78%)**	**0.61**	**-0.03 (-0.15 to 0.09)**
Secondary outcomes*						
Exclusive breastfeeding from childbirth to 6 weeks	105	47 (45%)	105	43 (41%)	0.58	0.04 (-0.10 to 0.17)
Any form of breastfeeding from childbirth to 6 weeks	105	98 (93%)	105	96 (91%)	0.60	0.02 (-0.05 to 0.09)
Exclusive breastfeeding from childbirth to 10 weeks	105	29 (28%)	102	24 (24%)	0.50	0.04 (-0.08 to 0.16)
Any form of breastfeeding from childbirth to 10 weeks	105	97 (92%)	102	91 (89%)	0.43	0.03 (-0.05 to 0.11)
All-cause child hospitalization or death	105	4 (2.9%)		9 (6.5%)	0.16	

*Exclusive breastfeeding and any form of breastfeeding from childbirth to each study visit based on 24-hour food recall interviews

The odds of exclusive breastfeeding at week 24 were 19% non-significantly lower in the intervention group compared to the standard care group, OR 0.81 (95% CI 0.26 to 2.51), *p* = 0.72.

Most mothers continued breastfeeding while adding other foods through week 24 (Tables 2 and 3). The intervention had no effect on any form of breastfeeding rates (75% versus 78%), rate difference − 3% (95% CI -15–9%) in complete case analysis and after inputting missing outcome data, -3% (95% CI -15–9%) (Table 3). The odds of any form of breastfeeding to week 24 were 15% non- significantly lower in the intervention group compared to the standard care group, OR 0.85 (95% CI 0.44 to 1.62), *p* = 0.61.

**Fig. 2:**
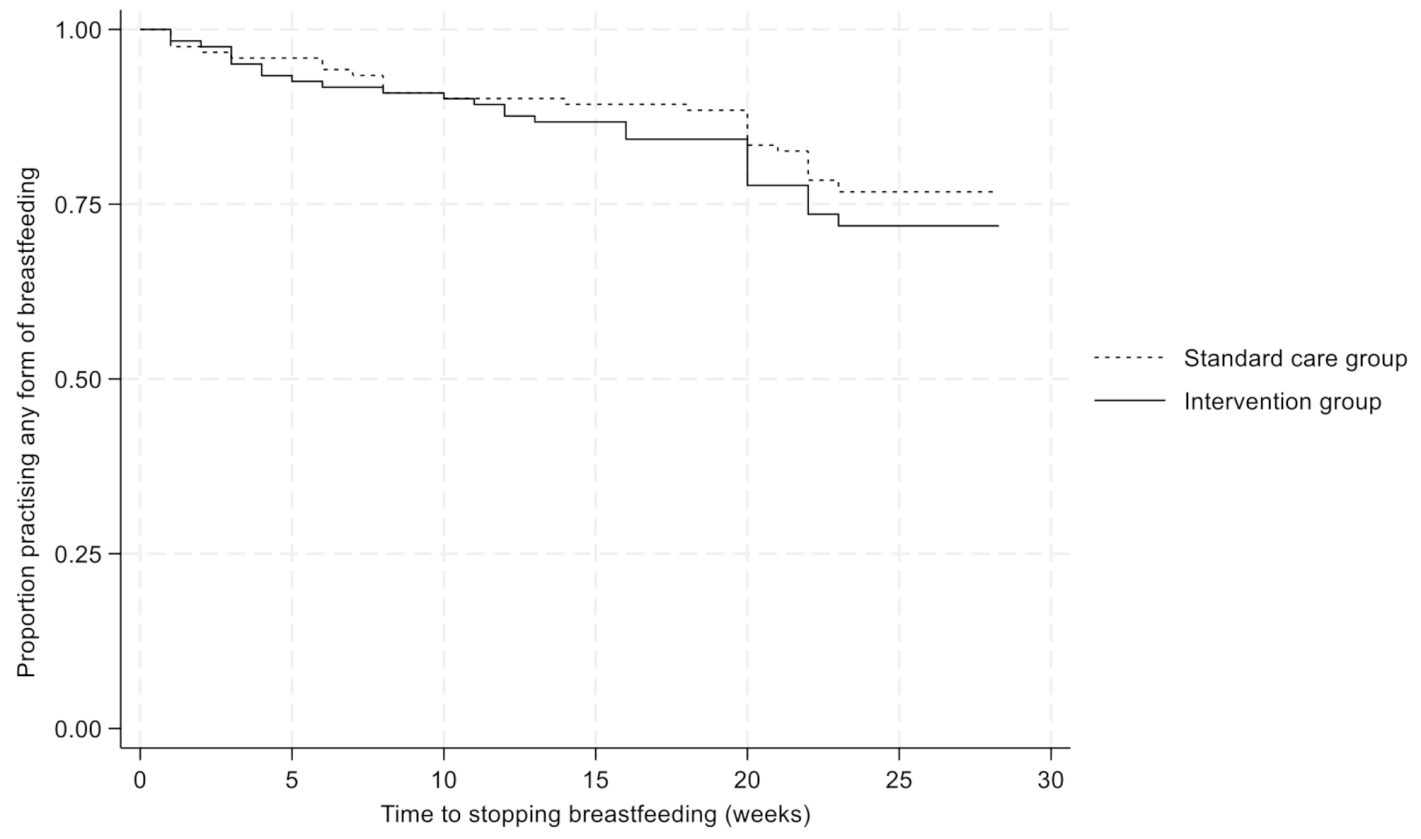
Time to stopping any form of breastfeeding, by study group

Sixty-two of 276 (22%) (34 (25%) in the intervention group and 28 (20%) standard care group) mother-child pairs completely stopped breastfeeding before week 24. The median (IQR) age at time of stopping breastfeeding was 16 (6 to 20) weeks. Time to stopping breastfeeding was similar in the study groups, *p* = 0.37, Fig. 2. The most common reasons reported by mothers for stopping breastfeeding were the mother needing to return to work or look for work, 66% (*n* = 41) and insufficient breastmilk or child refused breastmilk, 19% (*n* = 12).

### All-cause child hospitalization or death

**Fig. 3:**
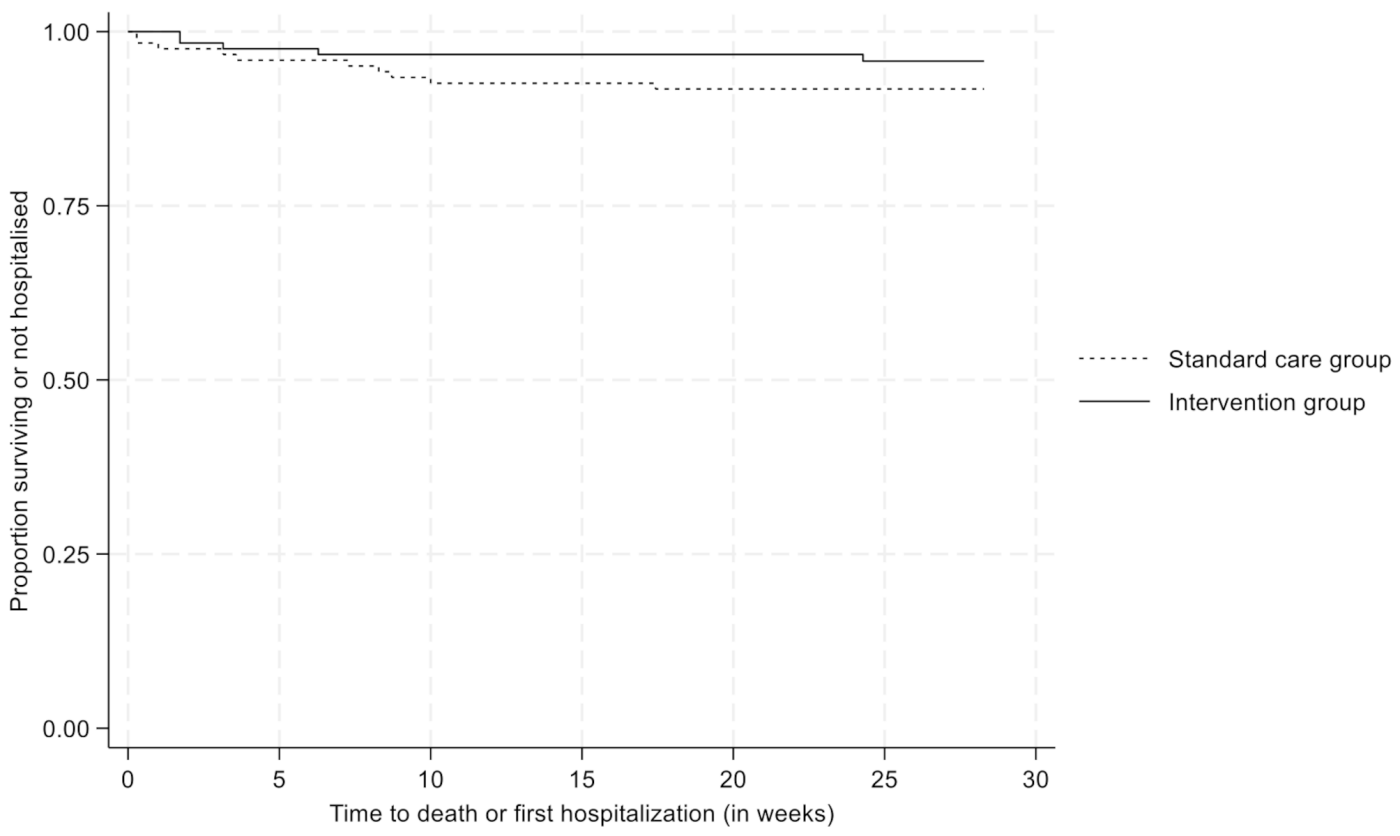
Time to death or first hospitalization, by study group

Thirteen children experienced 14 hospital admissions. Twelve children were each hospitalized once, and one child in the intervention group was hospitalized twice. Two hospitalized children died, one from each study group. The number of children hospitalized was not different between the intervention group and standard care group, 4 (2.9%) versus 9 (6.5%), *p* = 0.16. The intervention reduced the odds of hospitalization or death by 61%, OR 0.39 (95% CI 0.10 to 1.57), *p* = 0.19. Time to child death or first hospitalization was similar in the groups (Fig. 3), *p* = 0.18. Early breastfeeding cessation increased risk of child hospitalization or death compared to breastfeeding to 6 months, among children who completed all visits, 10% (5/48) versus 3% (5/158), *p* = 0.055. Early breastfeeding cessation more than tripled the odds of child hospitalization or death, OR 3.56 (95% CI 0.98 to 12.86).

Most children had diarrhea. Incidence of diarrhea decreased over time, rates were 91% at week 2, 86% at week 6, 63% at week 10 and 16% at week 24. Non-routine medically attended visits were similar; 29 in the intervention group and 30 for standard care.

### Infant growth

**Fig. 4:**
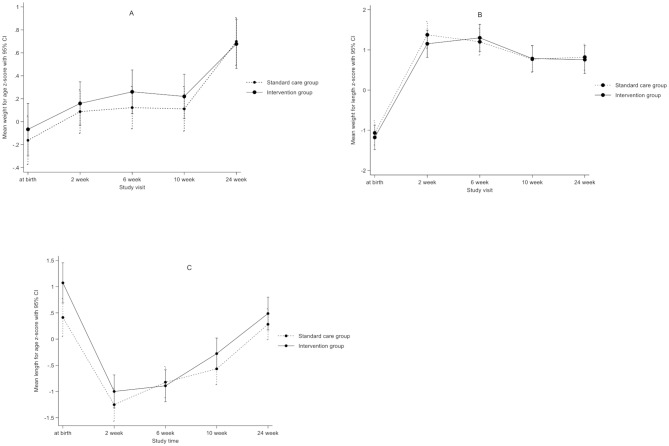
Mean weight for age z-score (**A**), weight for length z-score (**B**), length for age z-score (**C**), by study group

Infant growth was similar between study groups, (Fig. 4; Table 4). Mean weight for age z-scores increased over time in both groups (Fig. 4), with no significant difference between groups, *p* = 0.87. We found no significant difference in mean length for age z-scores, *p* = 0.21 and mean weight for length z-scores, *p* = 0.88.

**Table 4:** Infant growth outcomes, mean z-scores by study group

Study visit	Intervention	Standard care	Mean difference (95% CI)
**Mean weight for age z-score**			
Visit 2 (6 week)	0.26	0.12	0.14 ( -0.13 to 0.40)
Visit 3 (10 week)	0.22	0.11	0.11 (-0.16 to 0.38)
Visit 4 (24 week)	0.68	0.70	-0.02 ( -0.32 to 0.28)
**Mean length for age z-score**			
Visit 2 (6 week)	-0.89	-0.82	-0.07( -0.49 to 0.36)
Visit 3 (10 week)	-0.28	-0.57	0.29 ( -0.13 to 0.71)
Visit 4 (24 week)	0.49	0.28	0.21 ( -0.22 to 0.63)
**Mean weight for length z-score**			
Visit 2 (6 week)	1.30	1.20	0.10 ( -0.37 to 0.57)
Visit 3 (10 week)	0.78	0.77	0.01 ( -0.45 to 0.48)
Visit 4 (24 week)	0.76	0.82	-0.06 ( -0.54 to 0.41)

### Safety outcomes

Most mothers reported cART adherence in the month prior to each visit as very good or excellent across all visits, rates of very good or excellent adherence ranged between 90% and 97%. Study participation led to no involuntary disclosure of HIV status. No mother reported relationship conflicts with their partners due to study participation. Child immunization profiles were similar across study groups, all infants received BCG at birth, 99% received the week 6, 10 and 14 immunizations and only 19% had received the six-month immunizations.

## Discussion

Our primary objective was to demonstrate superiority of the intervention on sustaining exclusive breastfeeding to week 24 among mothers living with HIV in Western Cape Province, South Africa. However, we found no effect of motivational interviewing and text messaging compared to standard infant feeding counselling on exclusive breastfeeding or any form of breastfeeding rates at week 24. We however, found a marginal increase on exclusive breastfeeding rates in the intervention group through week 10, declining precipitously by week 24. The modest exclusive breastfeeding rates in early infancy within the standard infant feeding counselling group exceeded the 8% rate we initially expected. Our findings support a study reporting an increase in 4 to 8 weeks exclusive breastfeeding rates, coinciding with the Tshwane Declaration of support for breastfeeding [27]. Similarly, a cohort study found that South African mothers living with HIV breastfed exclusively in the first three weeks with a significant drop four months later [11]. By 12 months of age, approximately 40% of children born to mothers living with HIV in South Africa are breastfed [9].

A comparison between the findings of our cohort study 12 years ago and our current trial demonstrate an improvement from 50% breastfeeding cessation rate [28] to 22% in 2024. Additionally, there has been a marked increase in any form of breastfeeding practice over the past decade. The increase may be attributed to efforts at national and provincial levels to promote exclusive breastfeeding among mothers living with HIV, following adoption of the breastfeeding policy. However, early breastfeeding cessation remains a public health threat. Work-related demands influenced mothers’ decision to stop breastfeeding. Our findings showed that maternal employment characteristics are important social determinants of breastfeeding behaviour. While we report lack of effect of the intervention, various studies have shown that motivational interviewing and text messaging promoted weight loss, and improved medication adherence and retention in care among patients living with HIV [18, 19]. Mothers who were predominantly unemployed had socioeconomic competing demands affecting their breastfeeding practices that were not addressed by the intervention. We assume this contributed to the lack of effect of the intervention on exclusive breastfeeding practices. Maternal employment and schooling demands are key barriers to breastfeeding among mothers living with HIV [29]. To the best of our knowledge this is the first study that assessed the effect of motivation interviewing combined with text messaging on breastfeeding practices among socioeconomically disadvantaged mothers living with HIV. Motivational interviewing was beneficial across many health problems. We recommend future studies evaluating the effect of motivational interviewing in combination with financial incentives to address the socioeconomic needs of socio-economically disadvantaged mothers of reproductive and working age.

The common practice of any form of breastfeeding among mothers with high cART adherence contributed to low incidence of child hospitalization or death from any cause. The increased risk of child hospitalization or death among those who stopped breastfeeding emphasizes the benefits of any form of breastfeeding. This is consistent with other studies reporting an association between breastfeeding and infection-related hospitalization [9]. The findings affirm the WHO guiding practice statement “practicing mixed feeding is not a reason to stop breastfeeding in the presence of cART” [3]. Most mothers acquired HIV infection before pregnancy and had low HIV viral loads. Child hospitalization or death profiles are likely to be worse where mothers present with incident HIV infection, high viral load or poor cART adherence. While finding interventions to enhance exclusive breastfeeding remains critical, HIV services should reliably offer cART, consistently monitor viral load, and support mothers cART adherence, especially where mixed feeding is common. Mothers who stopped breastfeeding did not consider expressing breastmilk as an alternative to continue breastfeeding. There are opportunities during routine infant feeding counselling to educate mothers about social or work demands that may arise during breastfeeding and potential solutions to enable breastfeeding even during these challenges.

Our trial had limitations. We assessed exclusive breastfeeding up to 24 weeks as per the current recommendations. However, we acknowledge that at 24 weeks (± a few days) mothers are expected to start introducing complementary foods. Measuring exclusive breastfeeding rates at 24 weeks could have led to underestimation of the exclusive breastfeeding rates. We would recommend using an earlier timepoint e.g. 4 months or 5 months in future studies. The swift changes in length for age and weight for length z-scores from the first to the other visits could be due to errors in early length measurements.

## Conclusions

We found no effect of motivational interviewing plus text messaging on exclusive breastfeeding rates. Early cessation of breastfeeding was prevalent and maternal employment characteristics are important social determinants of breastfeeding behaviour. There is need for further research evaluating the effect of interventions that include financial incentives on breastfeeding practices among socioeconomically disadvantaged mothers. HIV services should reliably offer cART, consistently monitor viral load, and support mothers cART adherence, in settings where mixed feeding is common.

## Data Availability

The data will be deposited into the SUNScholarData, a data repository that is managed by Stellenbosch University library. Mothers had the option to consent or decline to their and their child’s anonymized and de-identified data being contributed to SUNScholarData and pooled with other maternal-child studies with appropriate institutional review board approvals for any additional pooled analyses.

## Declarations

### Authors’ information

List of study investigators: Prof Mark Cotton, Stellenbosch University; Dr Christine Els, Khayelitsha District Hospital; Prof Louise Kuhn, Columbia University; Prof Lehana Thabane, McMaster University; Prof Taryn Young, Stellenbosch University; Dr Moleen Zunza, Stellenbosch University. Independent data analyst: Dr Carl Lombard, Stellenbosch University. List of voting DSMB (Data Safety Monitoring Board) members: Prof Jonathan Levin (Chair), University of Witwatersrand, South Africa. Email: Jonathan.Levin@wits.ac.za; Prof Hassan Mahomed, Stellenbosch University, South Africa. Email: hmahomed@sun.ac.za; Prof Lawrence Mbuagbaw, McMaster University, Canada. Email: mbuagblc@mcmaster.ca; Prof Kennedy Otwombe, University of Witwatersrand, South Africa. Email: otwombek@phru.co.za; Dr Vundli Ramokolo, Medical Research Council, South Africa. Email: Vundli.Ramokolo@mrc.ac.za; Dr Elizabeth Spooner, Medical Research Council, South Africa. Email: Elizabeth.Spooner@mrc.ac.za.

## Abbreviations

cART: Combination antiretroviral treatment
WHO: World Health Organization
DSMB: Data Safety Monitoring Board
EDCTP: European & Developing Countries Clinical Trials Partnership
HEU: HIV exposed uninfected
HIV: Human immunodeficiency virus
PI: Principal investigator
RALLOC: Random number generating command
REDCap: Research Electronic Data Capture
CONSORT: Consolidated Standards of Reporting Trials

## References

[1] Victora CG, Bahl R, Barros AJD, França GVA, Horton S, Krasevec J, et al. Breastfeeding in the 21st century: epidemiology, mechanisms, and lifelong effect. Lancet. 2016;387(10017):475–90. 26869575 10.1016/S0140-6736(15)01024-7

[2] Rollins N, Bhandari N, Hajeebhoy N. Why invest, and what it will take to improve breastfeeding practices? Lancet. 2016;387(10017):491–504. 26869576 10.1016/S0140-6736(15)01044-2

[3] World Health Organization, United Nations Children’s Fund. Guideline: updates on HIV and infant feeding: the duration of breastfeeding, and support from health services to improve feeding practices among mothers living with HIV. Geneva: World Health Organization; 2016. 27583316

[4] Myer L, Phillips T. Beyond ‘Option B+’. J Acquir Immune Defic Syndr JAIDS. 2017;75(Suppl 2):S115–22. 28498180 10.1097/QAI.0000000000001343

[5] Wessels J, Sherman G, Bamford L. The updated South African National Guideline for the Prevention of Mother to Child Transmission of Communicable infections (2019). S Afr J HIV Med. 2020;21(1):a1079. 10.4102/sajhivmed.v21i1.1079 PMC7433286 32832113

[6] Kufa-Chakezha T, Shangase N, Lombard C, Manda S, Puren A. November. The 2022 Antenatal HIV Sentinel Survey Key Findings. https://www.nicd.ac.za/the-2022-antenatal-hiv-sentinel-survey. Accessed 16 2024.

[7] Issaka AI, Agho KE, Renzaho AMN. Prevalence of key breastfeeding indicators in 29 sub-saharan African countries: a meta-analysis of demographic and health surveys (2010–2015). BMJ Open. 2017;7(10):e014145. 29070635 10.1136/bmjopen-2016-014145 PMC5665288

[8] Remmert JE, Mosery N, Goodman G, Bangsberg DR, Safren SA, Smit JA, et al. Breastfeeding practices among women living with HIV in kwaZulu-Natal, South Africa: an observational study. Matern Child Health J. 2020;24(2):127–34. 31832911 10.1007/s10995-019-02848-8 PMC7311074

[9] le Roux S, Abrams E, Donald K, Brittain K, Phillips T, Zerbe A, et al. Infectious morbidity of breastfed, HIV-exposed uninfected infants under conditions of universal antiretroviral therapy in South Africa: a prospective cohort study. Lancet Child Adolesc Health. 2020;4(3):220–31. 31932246 10.1016/S2352-4642(19)30375-X PMC7235356

[10] Kimani-Murage EW, Wekesah F, Wanjohi M, Kyobutungi C, Ezeh AC, Musoke RN, et al. Factors affecting actualisation of the WHO breastfeeding recommendations in urban poor settings in Kenya. Matern Child Nutr. 2015;11(3):314–32. 25521041 10.1111/mcn.12161 PMC6860346

[11] Goga AE, Doherty T, Jackson DJ, Sanders D, Colvin M, Chopra M, et al. Infant feeding practices at routine PMTCT sites, South Africa: results of a prospective observational study amongst HIV exposed and unexposed infants - birth to 9 months. Int Breastfeed J. 2012;7:4. 22472507 10.1186/1746-4358-7-4 PMC3348038

[12] South African National Department of Health. Guidelines for Vertical Transmission Prevention of Communicable Infections 2023. https://knowledgehub.health.gov.za/elibrary/guideline-vertical-transmission-prevention-communicable-infections. Accessed 16 November 2024.

[13] Jones DL, Rodriguez VJ, Mandell LN, Lee TK, Weiss SM, Peltzer K. Influences on exclusive breastfeeding among rural HIV-infected South African women: cluster randomized control trial. AIDS Behav. 2018;22(9):2966–77. 29926300 10.1007/s10461-018-2197-z

[14] Iribarren S, Beck S, Pearce P, Chirico C, Etchevarria M, Cardinale D, Rubinstein F. TextTB: a mixed method pilot study evaluating acceptance, feasibility, and exploring initial efficacy of a text messaging intervention to support TB treatment adherence. Tuberc Res Treat. 2013;2013:349394. 24455238 10.1155/2013/349394 PMC3876704

[15] Dick J, Nundy S, Solomon M, Bishop K, Chin M, Peek M. Feasibility and usability of a text message-based program for diabetes self-management in an urban African-American population. J Diabetes Sci Technol. 2011;5(5):1246–54. 22027326 10.1177/193229681100500534 PMC3208889

[16] Pop-Eleches C, Thirumurthy H, Habyarimana JP, Zivin JG, Goldstein MP, de Walque D, et al. Mobile phone technologies improve adherence to antiretroviral treatment in a resource-limited setting: a randomized controlled trial of text message reminders. AIDS. 2011;25(6):825–34. 21252632 10.1097/QAD.0b013e32834380c1 PMC3718389

[17] Miller W, Rollnick S. Motivational interviewing: preparing people to change behaviour. New York: Guilford Press; 1991.

[18] Armstrong MJ, Mottershead TA, Ronksley PE, Sigal RJ, Campbell TS, Hemmelgarn BR. Motivational interviewing to improve weight loss in overweight and/or obese patients: a systematic review and meta-analysis of randomized controlled trials. Obes Rev. 2011;12(9):709–23. 21692966 10.1111/j.1467-789X.2011.00892.x

[19] Sukmaningrum E, Ayu AP, Wongso LV, Handayani M, Hendrianti S, Kawi NH, et al. Motivational interviewing as an intervention to improve antiretroviral treatment initiation among people who inject drugs (PWID): a pilot study in Jakarta and Bandung, Indonesia. Curr Drug Res Rev. 2024;16(2):228–36. 37259929 10.2174/2589977515666230531154629 PMC11340277

[20] Tylleskär T, Jackson D, Meda N, Engebretsen IMS, Chopra M, Diallo AH, et al. Exclusive breastfeeding promotion by peer counsellors in sub-saharan Africa (PROMISE-EBF): a cluster-randomised trial. Lancet. 2011;378(9789):420–7. 21752462 10.1016/S0140-6736(11)60738-1

[21] Chinkonde JR, Hem MH, Sundby J. HIV and infant feeding in Malawi: public health simplicity in complex social and cultural contexts. BMC Public Health. 2012;12:700. 22925437 10.1186/1471-2458-12-700 PMC3489588

[22] Odeny BM, Pfeiffer J, Farquhar C, Igonya EK, Gatuguta A, Kagwaini F, et al. The stigma of exclusive breastfeeding among both HIV-positive and HIV-negative women in Nairobi, Kenya. Breastfeed Med. 2016;11(5):252–8. 27093583 10.1089/bfm.2016.0014 PMC4921896

[23] Hazemba AN, Ncama BP, Sithole SL. Promotion of exclusive breastfeeding among HIV-positive mothers: an exploratory qualitative study. Int Breastfeed J. 2016;11:9. 27103938 10.1186/s13006-016-0068-7 PMC4839145

[24] Zunza M, Thabane L, Kuhn L, Els C, Cotton MF, Young T. Mobile phone text messaging plus motivational interviewing versus usual care: study protocol for a randomized controlled trial to evaluate effects on breastfeeding, child health, and survival outcomes, among women living with HIV (MTI-MI). Trials. 2023;24:639. 37794523 10.1186/s13063-023-07647-9 PMC10552370

[25] Schulz KF, Altman DG, Moher D. CONSORT 2010 Statement: updated guidelines for reporting parallel group randomised trials. BMJ. 2010;340:c332. 20332509 10.1136/bmj.c332 PMC2844940

[26] World Health Organization. Breastfeeding and replacement feeding practices in the context of mother-to-child transmission of HIV: an assessment tool for research and programs. Geneva: WHO; 2001.

[27] Jackson D, Swanevelder S, Doherty T, Lombard C, Bhardwaj S, Goga A. Changes in rates of early exclusive breastfeeding in South Africa from 201 to 2013: data from three national surveys before and during implementation of a change in national breastfeeding policy. BMJ Open. 2019;9(11):e028095. 31740463 10.1136/bmjopen-2018-028095 PMC6886934

[28] Zunza M, Esser M, Slograve A, Bettinger JA, Machekano R, Cotton M. Early Breastfeeding Cessation among HIVInfected and HIVUninfected women in Western Cape Province, South Africa. AIDS Behav. 2018;22(Suppl 1):114–20. 29959720 10.1007/s10461-018-2208-0 PMC6091631

[29] Nabulsi M. Why are breastfeeding rates low in Lebanon? A qualitative study. BMC Pediatr. 2011;11:75. 21878101 10.1186/1471-2431-11-75 PMC3175169

